# Daily Meaning in Life Buffers the Within‐Person Relationships Between Perceived Burdensomeness and Thwarted Belongingness With Next‐Day Suicidal Ideation: An Ecological Momentary Assessment Study

**DOI:** 10.1111/sltb.70136

**Published:** 2026-08-25

**Authors:** David P. Cenkner, Annie Duan, Adam G. Horwitz, Kamalakannan SO M Vijayakumar, Candice L. Odgers, Helen J. Burgess, Alyson K. Zalta

**Affiliations:** ^1^ Department of Psychology University of California, Irvine Irvine California USA; ^2^ Department of Psychiatry University of Michigan Medical School Ann Arbor Michigan USA; ^3^ Department of Psychiatry and Human Behavior University of California, Irvine Irvine California USA

**Keywords:** Bayesian analysis, dialectical behavior therapy, ecological momentary assessment, interpersonal theory of suicide, meaning in life, protective factors, suicide

## Abstract

**Introduction:**

The interpersonal theory of suicide posits that perceived burdensomeness and thwarted belongingness drive suicidal desire. Ecological momentary assessment studies have provided empirical support for these relationships. Although meaning in life protects against suicidal ideation, no research has examined its daily predictive or moderating effects on interpersonal theory variables and suicidal ideation.

**Methods:**

Thirty‐seven adults enrolled in a dialectical behavior therapy program were prompted to complete four assessments daily for 21 days, assessing suicidal ideation, perceived burdensomeness, and thwarted belongingness. Meaning in life was captured once daily. Multilevel models using frequentist and Bayesian frameworks tested within‐ and between‐person associations on next‐day suicidal ideation.

**Results:**

Meaning in life exhibited substantial daily variability. When participants reported greater daily meaning in life compared to their own average, there were weakened associations between perceived burdensomeness and thwarted belongingness with next‐day suicidal ideation, covarying for same‐day suicidal ideation.

**Conclusions:**

Results suggest daily meaning in life protects against next‐day suicidal ideation by buffering interpersonal theory variables. Meaning‐focused interventions targeting daily fluctuations in meaning in life may reduce acute suicide risk.

## 1 Introduction

Death by suicide in the United States is a public health crisis. In 2024, over 48,000 individuals in the United States died by suicide and approximately 14.3 million endorsed having serious thoughts of suicide (Substance Abuse and Mental Health Services Administration 2025). Individuals who experience suicidal thoughts have been found to have increased chances of having a psychological diagnosis, engage in a future suicide attempt, and use greater healthcare resources compared to those without suicidal thoughts (Jaffe et al. 2019; Nock et al. 2008; Reinherz et al. 2006). Therefore, examining risk factors that may lead individuals to experience suicidal thoughts is critical to identify potential intervention targets for suicide prevention.

Commonly studied risk factors for suicidal thoughts are perceived burdensomeness and thwarted belongingness, which come from the interpersonal theory of suicide (Joiner 2005; Van Orden et al. 2010). Perceived burdensomeness refers to believing one is a burden on others and that others would be better off without them. Thwarted belongingness refers to lacking connections with others, leading to loneliness or social isolation. A meta‐analysis examining 114 studies found support that perceived burdensomeness and thwarted belongingness were associated with suicidal ideation (Chu et al. 2017), yet most (92.3%) of the studies were cross‐sectional, highlighting the need for longitudinal research to understand the directionality of these associations.

Intensive longitudinal methodology involves frequent, repeated measurements, known as ecological momentary assessments (EMA), allowing examination of within‐person predictors of suicidal ideation longitudinally across days. Given that suicidal thoughts fluctuate within the same day (Coppersmith et al. 2023; Kleiman et al. 2017), EMA captures these fluctuations and reduces recall bias. EMA may likewise be critical to understanding acute factors that increase risk for, or protect against, the daily experience of suicidal thoughts. Prior EMA studies have demonstrated that within‐person perceived burdensomeness and thwarted belongingness concurrently and prospectively predict suicidal thoughts (Hallensleben et al. 2019; Jacobucci et al. 2023; Kleiman et al. 2017; Sels et al. 2024). Yet, few, if any, EMA studies have examined protective factors that may buffer the within‐day relationships between perceived burdensomeness and thwarted belongingness with suicidal thoughts.

Meaning in life may be one such protective factor. A systematic review indicated that meaning in life protected against suicidal thoughts, suicide attempts, and death by suicide (Costanza et al. 2019). Longitudinal studies from diverse samples found that meaning in life decreased suicidal thoughts over time (Heisel and Flett 2016; Kim et al. 2017; Kleiman and Beaver 2013). In a cross‐sectional study, purpose in life mediated the relationship between perceived burdensomeness and suicidal ideation, but not thwarted belongingness and suicidal ideation (Mora‐Ascó et al. 2025). In an 8‐week longitudinal study with two timepoints, meaning in life mediated the associations of perceived burdensomeness and thwarted belongingness with suicidal ideation (Kleiman and Beaver 2013). However, no study to date has used EMA to examine whether meaning in life might weaken the relationships between perceived burdensomeness and thwarted belongingness with suicidal ideation.

Given that meaning in life can be enhanced through psychotherapies such as logotherapy or acceptance and commitment therapy (Breitbart et al. 2015; Frankl 1992; Hayes et al. 2006; Pirfalak et al. 2024; Vos et al. 2015; Vos and Vitali 2018), it is critical to understand the daily variability of meaning in life and whether meaning in life is an acute protective factor against suicidal thoughts. The objectives of the current study were to: (1) examine the daily variability of meaning in life within individuals over time, (2) test whether on days when individuals experience greater meaning in life compared to their own average, they report lower next‐day suicidal ideation, and (3) examine whether daily meaning in life buffers the within‐person associations between perceived burdensomeness and thwarted belongingness with next‐day suicidal ideation. If found to protect against suicidal thoughts at the daily level, this may have clinical implications in how meaning is addressed in the therapeutic setting when working with individuals at high suicide risk.

## 2 Methods

### 2.1 Participants

Thirty‐seven adults enrolled in a 56‐day intensive outpatient program at a dialectical behavior therapy (DBT) treatment center participated in this study. Participants had a mean age of 33.11 (SD = 9.57), were majority White (68%), and female‐identifying (78%). Participants were able to enroll in the study at any point during their 56‐day treatment if they had 21 days (length of the EMA study) of treatment left. Eligible participants were required to be 18 years or older, English speaking, and own a smartphone to download the Avicenna app (http://avicennaresearch.com/). Adults enrolled in the DBT intensive outpatient program typically presented with emotion dysregulation and symptoms related to major depressive disorder, borderline personality disorder, post‐traumatic stress disorder, and/or endorsed current suicidal thoughts. Although a mental health diagnosis was not required for adults to enroll in the DBT treatment center, adults did have to undergo a clinical intake to determine suitability for the program.

### 2.2 Procedures

Data collection occurred at an adherent DBT treatment center in southern California. The DBT center operated three simultaneous 8‐week intensive outpatient programs where participants received individual therapy, phone coaching, and DBT group skills training. Each of the three intensive outpatient programs followed the same curricula, with one operating from 9 am to noon, one from 11 am to 2 pm, and one from 5 pm to 8 pm. Adults participating in any one of these three intensive outpatient programs were invited to participate in the study by their individual clinician. If interested, the participant was consented into the study. All assessments used the Avicenna app, with participants potentially earning up to $115 for full protocol completion. This study received institutional review board (IRB) approval through the University of California, Irvine (IRB #3323).

### 2.3 Ecological Momentary Assessment Schedule

Four daily EMA prompts were delivered pseudo‐randomly within designated 30 min to 1 h time blocks, with 1 h to respond to each prompt. Timing of the EMA surveys varied by intensive outpatient group based on the treatment schedule, so no EMA surveys were sent during the participant's intensive outpatient group. Participant schedules were adjusted if their wake and/or bedtimes were outside the standard EMA window.

### 2.4 Measures

During the first survey each day, the EMA question began with, “Since you awoke,” During the second, third, and fourth surveys, each question began with, “During the past 2 hours,” These prompts were selected to cover as much of the day as possible. The “past 2 hours” was chosen to decrease the possibility of data contamination if a participant missed an assessment. During all four surveys, participants were asked to rate their suicidal ideation duration, suicidal ideation intensity, perceived burdensomeness, and thwarted belongingness. Meaning in life was captured during the final survey of the day. The prompts for the EMA suicidal ideation duration and intensity, perceived burdensomeness, and thwarted belongingness questions were used in prior research (Czyz et al. 2023; Jiang et al. 2023; Parrish et al. 2021). The EMA prompt for the meaning in life question was adapted from the first question of the Meaning in Life Questionnaire (Steger et al. 2006). See Table 1 for the EMA questions and response choices.

**TABLE 1:** Ecological momentary assessment questions.

Variable	Question	Response scale
SI duration	Did you have any thoughts of suicide? If so, how long did these thoughts last?	0—Not at all/did not occur; 5–2 h/all the time
SI intensity	How strong or intense were these thoughts of suicide?	0—Not at all; 6—Extremely
Perceived burdensomeness	How much have you felt that you were a burden on others?	0—Not at all; 6—Extremely
Thwarted belongingness	How much have you been feeling like you belong or fit in with others?	0—Not at all; 6—Extremely
Meaning in life	Today, I understood my life's meaning.	0—Absolutely untrue; 6—Absolutely true

*Note:* SI duration, SI intensity, perceived burdensomeness, and thwarted belongingness were asked during all four daily surveys. Meaning in life was only asked during the final survey of the day. During the first survey each morning, the above questions began with, “Since you awoke” while the second, third, and fourth daily surveys began with, “During the past 2 hours.”

Abbreviation: SI, suicidal ideation.

### 2.5 Data Analytic Plan

**FIGURE 1:**
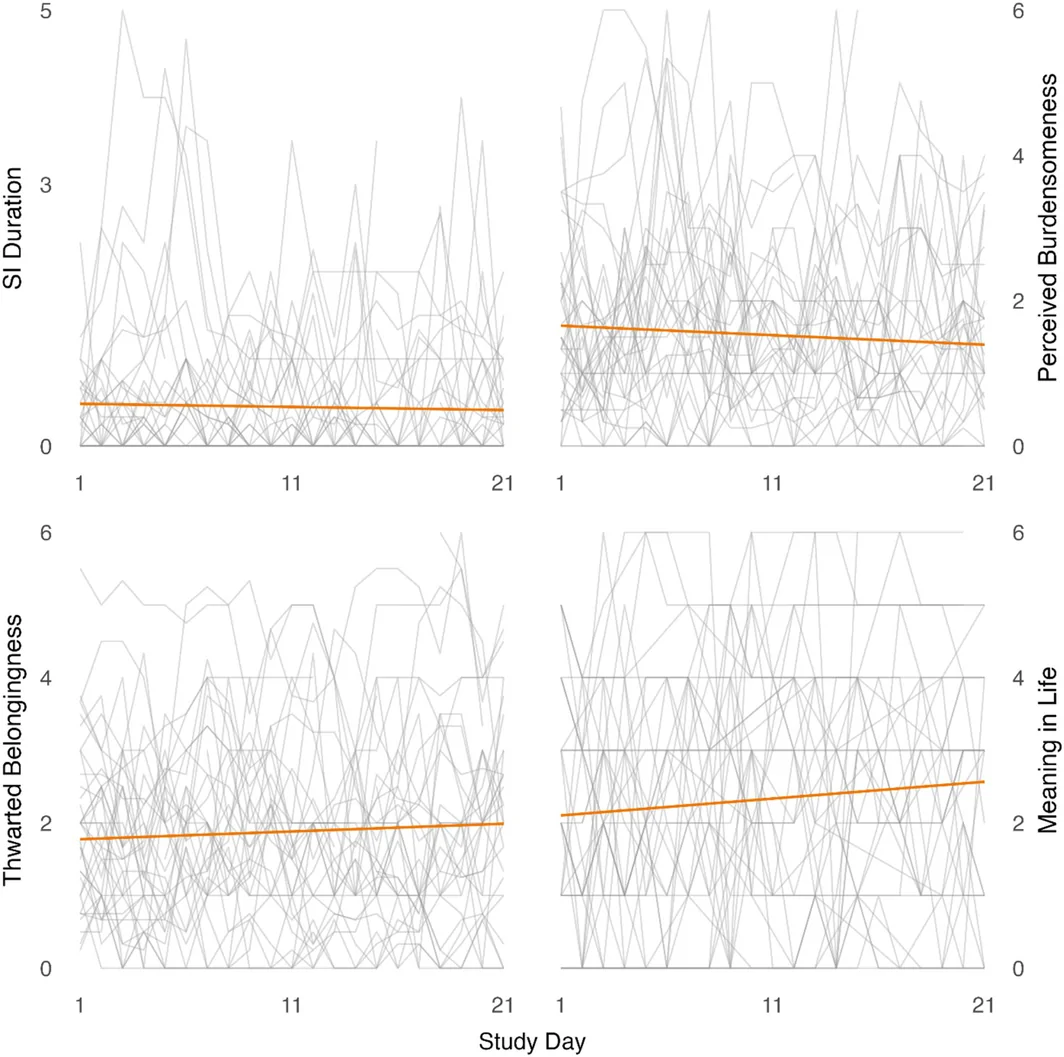
Spaghetti plots for the predictor and outcome variables. The solid orange line represents the regression line of each variable over the 21‐day study period. SI, suicidal ideation.

**FIGURE 2:**
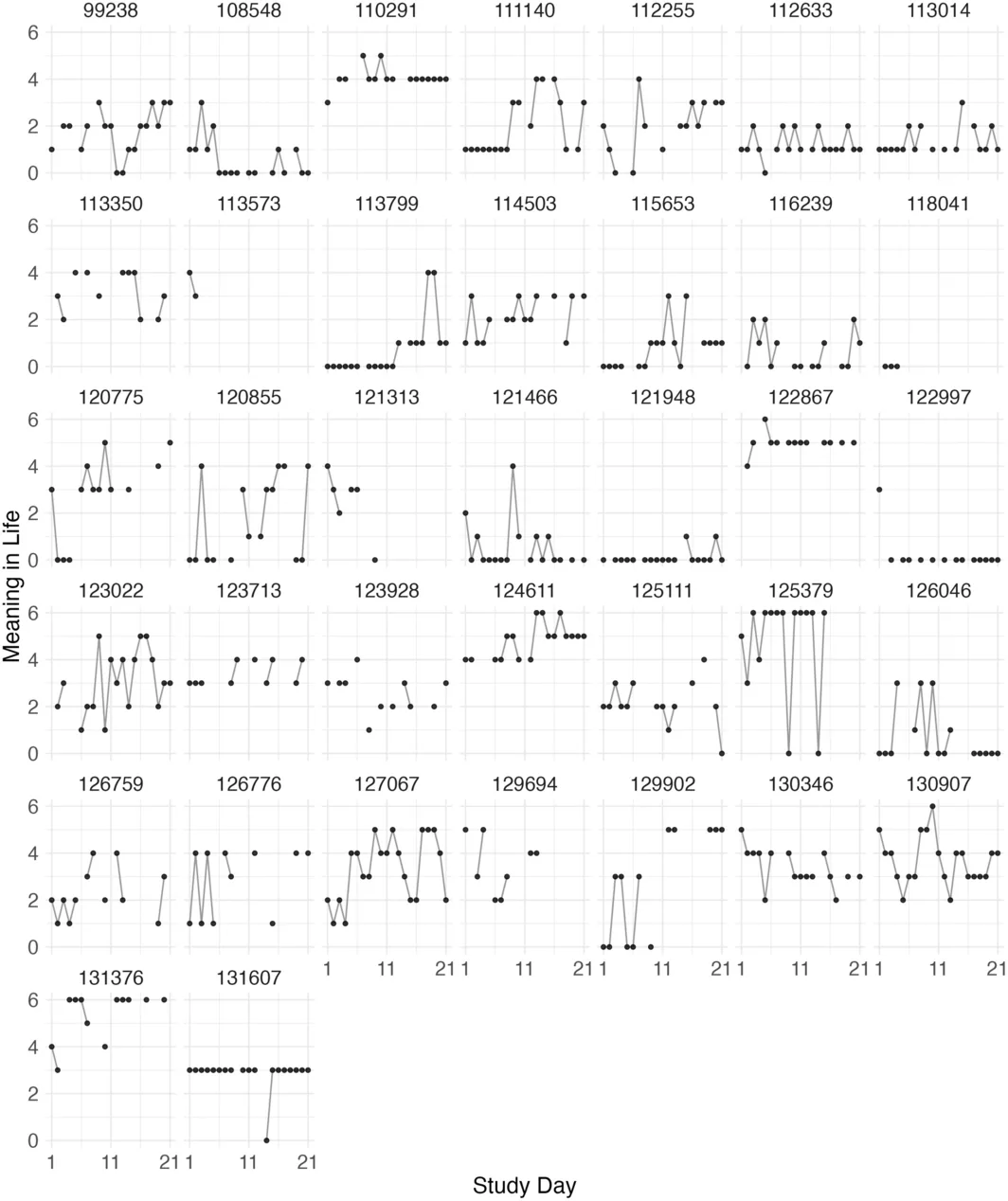
Individual participant day‐level responses to the daily meaning in life question. Individual black dots represent their day‐to‐day responses over the 21‐day study period. Line breaks across days represent missing data.

Duration of suicidal thoughts was selected as the primary outcome, with intensity examined as a sensitivity check. To first quantify the variability of each variable, the root mean square of successive differences (RMSSD) and the intraclass correlation coefficient (ICC) were calculated. RMSSD presents the average variability of a measure over time, with a larger RMSSD representing more variability between each point. The advantage of RMSSD is that it simultaneously accounts for the frequency, temporal dependency, and amplitude when conceptualizing instability (Ebner‐Priemer et al. 2009). ICCs present the proportion of between‐person variability and can range from zero to one. One minus the ICC represents the within‐person variability. Variability of all variables was visually captured using spaghetti plots (Figure 1), and raw individual participant panel plots were created to examine the within‐person variation of daily meaning in life (Figure 2).

Day‐level averages were created for perceived burdensomeness, thwarted belongingness, and suicidal ideation duration to represent individuals' overall daily experiences with these constructs. Day‐level averages were computed from whichever surveys a participant completed on a given day; therefore, a minimum of one completed survey was sufficient to generate a daily value. Meaning in life was assessed once daily during the final survey. This resulted in a two‐level data structure with days (Level 1) nested within participants (Level 2). Two‐level multilevel models were fit to test the study questions.

All predictor variables were person‐mean centered to disaggregate between‐ and within‐person components (Curran and Bauer 2011), then standardized. To test daily within‐person moderation, interaction terms were created by multiplying within‐person perceived burdensomeness with within‐person meaning in life (PB × MiL) and within‐person thwarted belongingness with within‐person meaning in life (TB × MiL). These interactions test whether on days when individuals experience greater meaning in life compared to their own average, the associations between perceived burdensomeness or thwarted belongingness and next‐day suicidal ideation are weakened. All analyses included random intercepts. To quantify the magnitude of moderation effects, a baseline model containing only main effects was compared to the full model including the perceived burdensomeness × meaning in life and thwarted belongingness × meaning in life interaction terms, and *R* ^2^ change (Δ*R* ^2^) was calculated.

Given that all participants were enrolled in an active DBT program during the study period, time in study (ranging from 1 to 21) was included as a covariate in all models. This allowed us to test whether daily meaning in life predicted suicidal ideation and moderated interpersonal theory variables above and beyond any linear treatment‐related changes that might have occurred over the 21‐day study period.

Due to the small sample size (*N* = 37), frequentist and Bayesian multilevel modeling approaches were used. Frequentist methods use power analyses based on alpha level, desired power, and assumed effect size. Bayesian methods do not rely on fixed significance thresholds. Instead, Bayesian methods generate posterior probability distributions capturing the probabilities of estimated parameter values. These two analytic frameworks use probabilities differently. Frequentist probability defines the likelihood of an event as its relative frequency across infinite hypothetical replications of an experiment under identical conditions. The frequentist method identifies the probability of the test statistic under the null hypothesis, and the null hypothesis is rejected if the probability is less than the prespecified alpha level. In contrast, Bayesian methods use probability in prior and posterior probability distributions to quantify uncertainty of parameter values. Bayesian inference combines prior information with observed data to produce a posterior distribution, which represents the updated plausibility of different parameter values.

Bayesian methods can offer advantages over frequentist approaches in certain settings. First, rather than producing a binary significant or nonsignificant decision based on a *p*‐value threshold, Bayesian estimation yields a full posterior probability distribution of the estimated parameter values conditional on the observed data (and prior), allowing for more nuanced interpretation of effects. Second, Bayesian estimation incorporates prior information through the prior probability distribution to help stabilize parameter estimates, which is particularly advantageous in studies with small sample sizes where frequentist estimates may be unstable or unreliable (Dora et al. 2024; Stegmueller 2013; Van De Schoot et al. 2015). A key feature of Bayesian estimation is the specification of prior distributions for model parameters including intercepts, slopes, and variance components. Incorporating informative priors offers particular advantages in EMA studies, which typically involve smaller sample sizes and large amounts of missing data. Prior specification can mitigate the risk of obtaining unstable or exaggerated parameter estimates (Dora et al. 2024; Stegmueller 2013; Van De Schoot et al. 2015). Third, Bayesian credible intervals (CrI) have a more direct and intuitive interpretation than frequentist confidence intervals. For example, the credible interval contains 95% of the posterior mass meaning that there is a 95% probability that the population parameter falls within the interval.

#### 2.5.1 Frequentist Analytic Plan

All analyses were conducted using Mplus version 8 (Muthen and Muthen 2017) with full information maximum likelihood to address missing data. To test the outcome of next‐day suicidal ideation, the between‐ and within‐person predictor variables of perceived burdensomeness, thwarted belongingness, meaning in life, their within‐person interactions, and time in study were entered into the model. Within‐person same‐day suicidal ideation was entered into the model as a covariate.

#### 2.5.2 Bayesian Analytic Plan

Analyses were conducted using the brms package (Bürkner 2017) in R 4.3.1. Parameter estimation used Markov chain Monte Carlo (MCMC) sampling across four independent chains, each running 2000 warm‐up samples followed by 4000 post warm‐up iterations for inference. Weakly informative priors were selected following best practices (Gelman 2006) to prevent implausible parameter values while allowing the data to drive the posterior estimates. Given that all predictors were *z*‐score standardized, parameter estimates were anticipated to range predominantly between −4 and 4. The intercept for next‐day suicidal ideation duration was assigned as normal (*M* = 0, SD = 1). Based on prior research (Chu et al. 2017), we expected positive slopes for both between‐person and within‐person perceived burdensomeness so these were assigned as normal (*M* = 1, SD = 1). Based on prior research (Chu et al. 2017), we expected negative slopes for both between‐person and within‐person thwarted belongingness because of how the question in the current study was worded so these were assigned as normal (*M* = −1, SD = 1). Further, the slopes of between‐person and within‐person meaning in life, the moderation of within‐person perceived burdensomeness and thwarted belongingness with meaning in life, and time in study were assigned normal (*M* = 0, SD = 1). All residual and random effect standard deviations were given exponential (1) priors.

## 3 Results

Descriptive statistics including missing data and ICCs for predictor and outcome variables are in Table 2. See Figure 1 for spaghetti plots of all variables. Seven participants did not endorse any suicidal ideation during the 21‐day study period and 17 (46%) engaged in a prior suicide attempt. Thirty‐four (91.9%) of participants completed equal to or greater than 70% of assessments over the 21‐day study period and 32 (86.5%) of participants responded to at least one of the four EMA daily surveys on their final (21st) study day.

**TABLE 2:** Descriptive statistics for predictor variables and suicidal ideation.

Variables	Range	Mean (SD)	*N*	ICC	*N* Day	% missing	RMSSD
Perceived burdensomeness	0–6	1.55 (0.89)	37	0.34	709	8.8	1.70
Thwarted belongingness	0–6	1.85 (1.05)	37	0.46	709	8.8	1.99
Meaning in life	0–6	2.38 (1.43)	37	0.62	533	31.4	2.29
Suicidal ideation duration	0–5	0.46 (0.51)	37	0.36	710	8.6	1.07

*Note:* Perceived burdensomeness, thwarted belongingness, and suicidal ideation were asked during all four daily surveys and averaged within 1 day. Meaning in life was only asked during the final survey each day. Total number of possible observations was 777 days. ICCs presented are from the item level variables of perceived burdensomeness, thwarted belongingness, and suicidal ideation duration, not their daily averages. *N* Day represents the number of days of data available for each variable.

Abbreviations: ICC, intraclass correlation; RMSSD, root mean square of successive differences.

### 3.1 Daily Variability of Meaning in Life

Based on the RMSSD of meaning in life (2.29), there was substantial day‐to‐day variability across participants. Further, the ICC of meaning in life (0.62) suggested that 38% of the variability was attributable to the within‐person level. To visually highlight the variability of meaning in life scores within each participant, individual panel plots were created (Figure 2).

### 3.2 Next‐Day Suicidal Ideation Duration

Table 3 presents the multilevel model results for the next‐day suicidal ideation outcome from both frequentist and Bayesian analyses. Between‐person results found that across the 21‐day study, participants who reported higher average levels of perceived burdensomeness also experienced higher average levels of suicidal ideation (*β*_Freq_ = 0.27, *p* < 0.001, 95% CI [0.11, 0.43]). Between‐person thwarted belongingness and meaning in life were not significant.

**TABLE 3:** Multilevel models examining the relationships between perceived burdensomeness, thwarted belongingness, meaning in life, and their interactions, with next‐day suicidal ideation.

Next‐day suicidal ideation	Frequentist results				Bayesian results	
Fixed effects	*β*	*t*	*p‐*value	95% CI	*β*	95% CrI
Intercept	0.47	5.91	< 0.001	0.31, 0.62	0.44	0.28, 0.59
PB within	−0.02	−0.54	0.590	−0.07, 0.04	−0.01	−0.07, 0.04
TB within	0.00	0.11	0.913	−0.05, 0.06	0.00	−0.05, 0.05
MiL within	−0.04	−1.76	0.078	−0.09, 0.01	−0.04	−0.09, 0.01
SI within	0.14	5.54	< 0.001	0.09, 0.19	0.14	0.09, 0.19
PB × MiL	−0.09	−4.27	< 0.001	−0.13, −0.05	−0.09	−0.13, −0.05
TB × MiL	−0.05	−2.13	0.033	−0.10, −0.00	−0.05	−0.10, −0.00
PB between	0.27	3.28	0.001	0.11, 0.43	0.28	0.09, 0.44
TB between	0.12	1.05	0.293	−0.10, 0.34	0.10	−0.14, 0.34
MiL between	−0.18	−1.44	0.151	−0.42, 0.07	−0.16	−0.42, 0.11
Time in study	−0.00	−1.00	0.315	−0.01, 0.00	−0.02	−0.07, 0.02

Random effects	Estimate (SD)	*z*	*p*‐value	95% CI	Estimate	95% CrI
Intercept variance (*τ*_00_)	0.17 (0.41)	3.88	< 0.001	0.08, 0.25	0.45	0.35, 0.58
Residual variance (*σ* ^2^)	0.21 (0.46)	15.14	< 0.001	0.18, 0.24	0.46	0.44, 0.50

*Note:* Frequentist random effects are reported as variance with the standard deviation (SD) in parentheses. Bayesian random effects are reported as posterior mean SD. Next‐day suicidal ideation number of observations = 495. All estimates are from standardized predictor variables. Moderator variables were created using within‐person variables. The thwarted belongingness scale is positively worded such that higher scores reflect greater belongingness. Therefore, the negative coefficients for thwarted belongingness and its interaction with meaning in life reflect effects in the theoretically expected direction.

Abbreviations: CI, confidence interval; CrI, credible interval; MiL, meaning in life; PB, perceived burdensomeness; SI, suicidal ideation; TB, thwarted belongingness.

**FIGURE 3:**
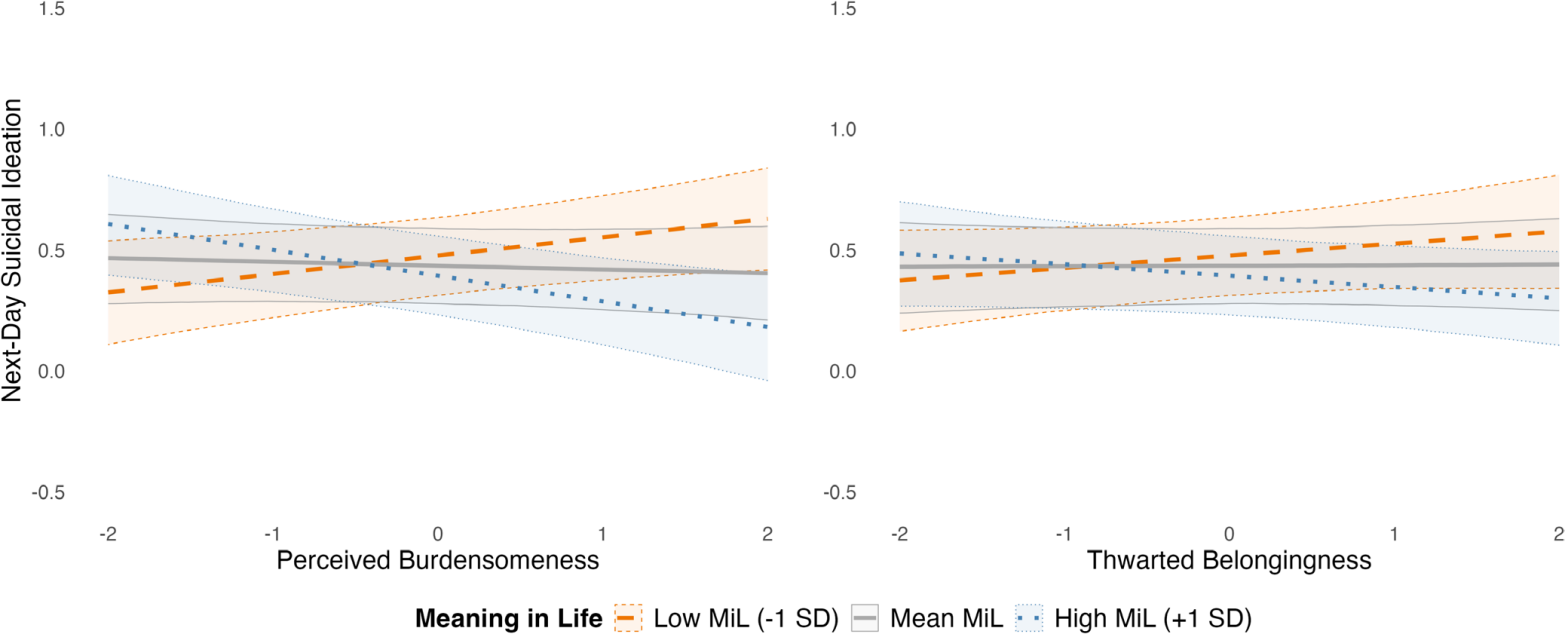
Bayesian moderation plots showing within‐person meaning in life buffering the relationships between perceived burdensomeness and thwarted belongingness with next‐day suicidal ideation. Lines represent median posterior predictions with shaded regions showing 95% credible intervals.

Within‐person results found no significant associations at the daily level between perceived burdensomeness or thwarted belongingness with next‐day suicidal ideation. A marginally significant finding was found for meaning in life such that on days when participants reported higher meaning in life compared to their own average, they were more likely to experience less next‐day suicidal ideation (*β*_Freq_ = −0.04, *p* = 0.078, 95% CI [−0.09, 0.01]), covarying for same‐day suicidal ideation (though see Bayesian results below regarding this relationship). Further, on days when participants reported higher suicidal ideation compared to their own average, they were more likely to experience higher next‐day suicidal ideation (*β*_Freq_ = 0.14, *p* < 0.001, 95% CI [0.09, 0.19]). Regarding the within‐person moderators, on days when participants experienced greater meaning in life compared to their own average, the association between daily perceived burdensomeness and next‐day suicidal ideation was significantly weakened (*β*_Freq_ = −0.09, *p* < 0.001, 95% CI [−0.13, −0.05]; Figure 3), covarying for same‐day suicidal ideation. When participants experienced greater meaning in life compared to their own average, the association between daily thwarted belongingness and next‐day suicidal ideation was significantly weakened (*β*_Freq_ = −0.05, *p* = 0.033, 95% CI [−0.10, −0.00]; Figure 3), covarying for same‐day suicidal ideation. The within‐person model accounted for 17% of the variance in the next‐day suicidal ideation outcome (*R* ^2^ = 0.17, *p* < 0.001).^1^ The addition of the perceived burdensomeness and thwarted belongingness interaction terms with meaning in life explained an additional 2% of variance beyond a baseline main‐effects only model (Δ*R* ^2^ = 0.02).

Bayesian results were largely consistent with frequentist results, though one result is particularly noteworthy for potential clinical significance. On days when participants reported higher meaning in life compared to their own average, they experienced lower next‐day suicidal ideation (*β*_Bayes_ = −0.04, 95% CrI [−0.09, 0.01]), covarying for same‐day suicidal ideation. The corresponding frequentist model found this not to be statistically significant (*β*_Freq_ = −0.04, *p* = 0.078). However, 96% of the posterior distribution fell below zero, indicating strong probabilistic evidence for a protective effect that the study may have been underpowered to detect using conventional frequentist statistical thresholds.

## 4 Discussion

The current study was the first to capture daily fluctuations of meaning in life among adults in a DBT intensive outpatient program. Further, this study was the first to test the role of meaning in life at the daily level as both a direct protective factor and moderator of interpersonal theory of suicide variables in next‐day suicidal ideation. Bayesian methods were employed to stabilize parameter estimates given the modest sample size and to provide posterior probability distributions for effects approaching conventional significance thresholds. Results suggest that cultivating daily meaning in life may be a promising intervention target for acute suicide risk reduction.

Meaning in life exhibited substantial day‐to‐day variability, with 38% of the differences in meaning occurring within the same person from day‐to‐day, rather than being stable differences between people. On average, participants' daily meaning in life scores changed by more than two points from 1 day to the next on a zero through six Likert scale. These findings suggest that meaning in life functions as a dynamic, state‐like construct rather than a stable trait, one that shifts meaningfully from day‐to‐day.

The variability in meaning in life is particularly noteworthy given that participants were enrolled in an intensive DBT program during the study period. Participants received 12 h of weekly group therapy and 1 h of individual therapy. Despite this intensive therapeutic environment, meaning in life still fluctuated substantially from day‐to‐day, suggesting that it is responsive to daily experiences and mood states even during active treatment. We found no significant effect of time in study, indicating that meaning in life did not systematically increase or decrease across the 21‐day study period. This suggests that the observed fluctuations reflect genuine day‐to‐day variability rather than a linear treatment effect, though longer assessment periods or larger samples might detect gradual improvements as DBT treatment progresses.

Clinically, this day‐to‐day variability is encouraging because it suggests meaning in life is malleable and potentially responsive to daily intervention. If meaning in life were primarily a stable trait, temporary interventions would be less likely to produce meaningful change. Instead, the observed daily fluctuations indicate that brief, targeted interventions could enhance meaning in life acutely, potentially offering protection during periods of elevated suicide risk. One explanation for this variability may be positive affect, which has been found to be associated with and predictive of increased meaning in life at both cross‐sectional and momentary levels (King et al. 2006; Lo et al. 2025). Future research examining the within‐person antecedents of daily meaning in life, which may include mood, social connection, religiosity or spirituality, or specific therapeutic experiences during DBT sessions, would help clarify the mechanisms driving this variability and identify additional intervention targets. Future research could also use qualitative or mixed‐methods to understand how individuals in acute suicidal distress conceptualize and derive meaning, which would help inform idiographic interventions.

The relationship between daily meaning in life and next‐day suicidal ideation was not statistically significant in the frequentist analysis (*p* = 0.078) after covarying for same‐day suicidal ideation. However, the Bayesian analysis revealed that 96% of the posterior distribution fell below zero, indicating strong probabilistic evidence for a protective effect. This suggests a possible carry over effect in which prior‐day meaning in life may influence how long suicidal thoughts last the following day. In the frequentist framework, this marginal finding might be dismissed as nonsignificant, but the Bayesian result suggests that the effect may carry clinical significance and warrants replication in larger samples.

Daily meaning in life significantly buffered the relationship between perceived burdensomeness and next‐day suicidal ideation. This buffering effect operated at the within‐person level such that on days when individuals experienced greater meaning in life compared to their own average, the association between perceived burdensomeness and next‐day suicidal ideation weakened, covarying for same‐day suicidal ideation. This result reveals that meaning in life operates as a dynamic, daily protective factor. One interpretation of these findings is that meaning in life may counteract the cognitive narrative underlying perceived burdensomeness which is the belief that one is a burden to others, by providing an alternative framework through which individuals can understand their value and place in the world. This mechanism may be particularly relevant in a DBT treatment context, where clients are explicitly working to challenge maladaptive cognitions and build lives worth living. Meaning in life may offer a cognitive reframe that allows individuals to evaluate their worth through purpose and contribution. This aligns with prior cross‐sectional work finding that purpose in life mediated the relationship between perceived burdensomeness and suicidal ideation (Mora‐Ascó et al. 2025) and extends it by demonstrating this buffering effect at the daily level in a clinical sample actively engaged in treatment.

The finding that meaning in life weakened the relationship between thwarted belongingness and next‐day suicidal ideation should be interpreted cautiously. Given that the effect size was small and that the *p*‐value approached equaling 0.05, it is possible that this represents a Type I error. Alternatively, the convergence of small negative point estimates and Bayesian credible intervals that touched but did not cross zero suggests that a small but potentially clinically meaningful buffering effect of meaning in life on the thwarted belongingness and next‐day suicidal ideation relationship may exist. Replication in larger samples is needed to clarify whether this moderation reflects a robust effect or a spurious finding.

Findings from the current study have clinical implications for psychological interventions aimed at treating individuals with suicidal thoughts. While DBT incorporates “life worth living goals” as part of treatment, existential meaning is not systematically targeted as a primary mechanism that may decrease suicidal thoughts. Our results suggest that focusing on daily meaning could enhance the effectiveness of DBT to reduce suicidal thoughts. Specifically, the substantial day‐to‐day variability of meaning in life suggests that brief, session‐by‐session discussion focused on sources of meaning, clarifying values, and connecting daily activities to broader life purpose may enhance acute protective benefits against suicidal thoughts.

Several evidence‐based approaches could be integrated into DBT to enhance meaning in life. Logotherapy (Frankl 1992) emphasizes acceptance of one's current position and finding meaning through suffering, a framework that aligns naturally with DBT's dialectical approach of accepting current reality while working toward change. Logotherapy techniques such as de‐reflection (shifting focus from internal distress to meaningful external pursuits) and Socratic dialogue could be adapted to brief daily exercises. Similarly, acceptance and commitment therapy and other meaning‐centered psychotherapies (Breitbart et al. 2015; Hayes et al. 2006; Pirfalak et al. 2024) have demonstrated effectiveness in cultivating meaning and reducing psychological distress. Further, the moderation findings suggest that interventions could be most effective when targeting meaning‐making in individuals experiencing high levels of perceived burdensomeness. Smartphone‐delivered EMA surveys could assess daily perceived burdensomeness and trigger logotherapy‐ or ACT‐based informed exercises when burdensomeness exceeds a threshold. This type of just‐in‐time intervention could help enhance meaning to attenuate acute suicide risk, capitalizing on the demonstrated buffering effect of meaning on the perceived burdensomeness to suicidal ideation relationship.

The current study has limitations. First, all participants were treatment‐seeking adults from a single DBT treatment center, thus limiting generalizability of our findings. Second, meaning in life was only asked during the final survey each day, resulting in more missing data compared to perceived burdensomeness, thwarted belongingness, and suicidal thoughts, which were asked during all four daily surveys. This prevented examining within‐day fluctuations of meaning in life. Third, the sample was predominantly White (68%) and female‐identifying (78%), limiting generalizability to more diverse populations. Research suggests that suicide risk factors, including perceived burdensomeness and thwarted belongingness, may operate differently across gender and racial/ethnic groups (Chu et al. 2017). Future research should examine whether daily meaning in life exerts similar protective effects in more diverse samples, and among males, who are disproportionately represented in suicide mortality despite lower rates of suicidal ideation. Fourth, the sample size in the current study was small. Small samples increase Type I error risk in frequentist analyses and uncertainty in Bayesian analyses.

Despite these limitations, this study makes several novel contributions. It was the first to examine the daily fluctuations of meaning in life in a clinical sample receiving intensive treatment for suicide risk, test its direct protective effect against next‐day suicidal ideation, and demonstrate that meaning in life moderates the relationship between interpersonal theory variables and next‐day suicidal thoughts at the daily level. Future research should aim to replicate these findings in larger, more diverse samples across different treatment settings. Studies may want to assess meaning in life more frequently to capture within‐day fluctuations and examine how meaning responds to specific therapeutic interventions or daily events. Qualitative methods or mixed‐methods would also be useful in understanding how individuals in acute suicidal distress conceptualize and derive meaning in their lives, which would help inform idiographic interventions. Additional research aiming to understand if meaning in life also offers near‐term protection against suicide attempts, and not just suicidal ideation, would be valuable. Finally, the use of Bayesian methods affords a greater holistic understanding of the relationships between the studied variables, rather than only showing whether these relationships were significant or not significant. Meaning in life is a promising, malleable target for daily intervention that may meaningfully reduce acute suicide risk.

## Author Contributions

**Alyson K. Zalta:** conceptualization, funding acquisition, methodology, resources, supervision, writing – review and editing. **Annie Duan:** investigation, project administration, writing – review and editing. **Kamalakannan SO M Vijayakumar:** formal analysis, software, writing – review and editing. **Candice L. Odgers:** supervision, writing – review and editing. **Adam G. Horwitz:** conceptualization, supervision, writing – review and editing. **Helen J. Burgess:** supervision, writing – review and editing. **David P. Cenkner:** conceptualization, formal analysis, funding acquisition, investigation, methodology, project administration, software, supervision, visualization, writing – original draft.

## Funding

This project was supported by a COGDOP scholarship from the American Psychological Foundation and the University of California, Irvine Community Engaged Research Fellowship.

## Ethics Statement

This study received institutional review board approval through the University of California, Irvine (IRB #3323).

## Consent

All participants provided written informed consent prior to participation.

## Conflicts of Interest

H.J.B. is a consultant for Natrol LLC.

## Data Availability

The data that support the findings of this study are available on request from the corresponding author. The data are not publicly available due to privacy or ethical restrictions.

## Endnotes

^1^
All models were repeated examining next‐day suicidal ideation intensity as the outcome, covarying for same‐day suicidal ideation intensity. Substantively, most results between the next‐day duration and intensity outcomes did not differ with one exception. Meaning in life did not significantly moderate the relationship between thwarted belongingness and next‐day suicidal ideation intensity (*β* = −0.04; *p* = 0.109).
